# Genetic Features of Uveal Melanoma

**DOI:** 10.3390/genes15111356

**Published:** 2024-10-22

**Authors:** Francesco Saverio Sorrentino, Carola Culiersi, Antonio Florido, Katia De Nadai, Ginevra Giovanna Adamo, Francesco Nasini, Chiara Vivarelli, Marco Mura, Francesco Parmeggiani

**Affiliations:** 1 Unit of Ophthalmology, Department of Surgical Sciences, Ospedale Maggiore, 40100 Bologna, Italy; dr.fsorrentino@gmail.com (F.S.S.); c.carola@live.it (C.C.); antonioflorido@outlook.it (A.F.); 2 Department of Translational Medicine and for Romagna, University of Ferrara, 44100 Ferrara, Italy; katia.denadai@unife.it (K.D.N.); ginevragiovanna.adamo@unife.it (G.G.A.); chiara.vivarelli@unife.it (C.V.); marco.mura@unife.it (M.M.); 3 ERN-EYE Network—Center for Retinitis Pigmentosa of Veneto Region, Camposampiero Hospital, 35100 Padova, Italy; 4 Unit of Ophthalmology, Azienda Ospedaliero Universitaria di Ferrara, 44100 Ferrara, Italy; francesco.nasini@ospfe.it; 5 King Khaled Eye Specialist Hospital, Riyadh 12211, Saudi Arabia

**Keywords:** uveal melanoma, genetic analysis, metastatic risk prediction

## Abstract

Background/Objectives: Although it comprises only 5% of all melanomas, uveal melanoma (UM) is the most commonly observed primary intraocular cancer. Methods: Poor patient survival persists in spite of innovative systemic therapies. In fact, approximately fifty percent of UM patients develop metastases from micro-metastases that remain undetected at the exact time of diagnosis. Results: The molecular understanding of UM is significantly enhanced by the recent identification of several mutations that are responsible for the metastasis, growth, and survival of UM. The crucial point is a more accurate genetic analysis for patient follow-up and metastatic risk prediction. Conclusions: This review provides a brief summary of the molecular features of UM that are recently discovered, as well as cytogenetic markers and biochemical pathways that are associated with the development of UM metastases.

## 1. Introduction

Uveal melanoma (UM) is the predominant primary intraocular malignancy in adults, constituting approximately 53.8% of all melanomas, with a particular impact on the Caucasian demographic [1]. Genetic heritage could have an impact on its development [2]. Despite being extremely rare, the prognosis is often poor due to a high risk of liver metastases and a lack of suitable treatment choices [3,4]. The clinical course of UM is quite distinctive from that of other types of melanoma, especially in terms of the response to systemic therapies and patterns of metastasis. Uveal melanoma primarily originates from the choroid, with less frequent occurrences in the eye and ciliary body. Mutations in BAP1, PLCβ4, GNAQ/11, and CYSLTR2 have been identified in a unique molecular landscape [5]. The subclassification of UMs arises from molecular findings, which have substantial prognostic implications and are crucial for patient care. Signaling pathways such as mTOR, JAK/STAT, and β-catenin are critical for carcinogenesis and development in many malignancies [6,7,8]. Understanding the immunological milieu and molecular frameworks enables the development of innovative treatment strategies [9]. Concurrently, innovative therapeutic approaches, such as tailored treatments and immunotherapies, are creating new possibilities for personalized care [10]. The therapy of metastatic uveal melanoma remains a considerable difficulty. This review aims to deliver a thorough assessment of the existing knowledge on UM, including its molecular etiology and diagnostic and prognostic biomarkers [11]. Furthermore, we examine the existing problems in this oncological domain and suggest prospective avenues for clinical management and research, aiming to improve the clinical outcomes for patients with UM.

## 2. Methods

A comprehensive literature search was conducted to identify relevant studies on genetic changes in UM. The databases searched included Scopus, Web of Science, and PubMed. Keywords included “uveal melanoma”, “genetic analysis”, and “metastatic risk prediction”. Articles were selected based on research focused on uveal melanoma, including peer-reviewed publications that included research articles, reviews, and clinical trials, as well as studies addressing ocular melanoma, all in the English language. The exclusion criteria were predicated on the irrelevance of studies to uveal melanoma, research focused solely on brachytherapy, abstracts, conference proceedings, and non-peer-reviewed publications.

The search approach employing general keywords yielded a substantial quantity of literature concerning the genetics of UM. Relevance was evaluated by examining the titles and abstracts of the selected publications. The complete texts of the shortlisted articles were scrutinized to ensure they met the criteria for inclusion. The reference lists of select papers were scrutinized to identify more relevant research. The study’s methodological rigor was assessed by considering the statistical analysis, experimental design, and sample size. A comprehensive summary of the genetic changes in UM was generated by synthesizing the collected data. The synthesis focused on a comprehensive investigation of modified genes in the context of uveal malignancies.

## 3. The Genetic Framework

The evolution and advancement of UM are defined by a distinct microenvironment influenced by a diverse range of genetic, cellular, and molecular modifications. Particular inherited genetic mutations, Caucasian heritage, flawless skin, exposure to UV radiation, and light-colored irises are all variables that can increase the risk of UM [12,13]. The three uveal clinic entities that exhibit distinctive clinical and genetic characteristics are choroidal melanomas, ciliary bodies, and iris melanomas [14]. In contrast to cutaneous melanomas, which have a higher tumor mutational burden, most uveal melanomas arise from the choroid, an eye tissue shielded from sunlight, resulting in a diminished mutational frequency [15,16]. Early mutations in genes, for instance, GNAQ/11, CYSLTR2, and PLCβ4 (known as “initiating mutations”), are the primary cause of UM. These mutations are followed by later mutations (known as “prognostic mutations”) in genes such as MAPKAPK5, EIF1AX, SF3B1, and SRSF2 (Table 1). Moreover, mutations in BAP1, a recognized tumor suppressor, substantially enhance the development of metastases by inactivating the protein, and the prognosis is severe [17,18]. BRAF- and NRAS-activating mutations are few or absent in uveal melanoma, unlike cutaneous melanoma [19]. Nonetheless, the T1799A point mutation in BRAF was identified in UM utilizing specialized techniques [20]. The clinical management of UM is greatly improved by the classification of patients into distinct prognosis clusters.

This classification guides therapeutic choices and patient eligibility for clinical trials.

Multiple variables, such as location, tumor volume, mutations, chromosomal rearrangements, and gene expression profile (GEP), are assessed by prognostic instruments that are currently in use [21,22]. For instance, UMs can be pigeonholed into three different subtypes on the basis of the expression of twelve discriminating mRNA transcripts (DecisionDx-UM GEP test): class 1A (2% five-year metastatic risk; low risk = low-intensity management with image-based surveillance every 12 months), class 1B (21% five-year metastatic risk; intermediate risk = moderate-intensity management with image-based surveillance every 6–12 months), and class 2 (72% five-year metastatic risk), which features an aggressive nature and fatal progression to metastatic disease (high risk = high-intensity management with image-based surveillance every 3–6 months and discussion for preventive treatment or clinical trial opportunities) [23]. Through the years, patients with UM have been classified into four molecularly and clinically different subgroups (1–4 or A–D) [24]. The more favorable prognosis categories include Class 1 or A, characterized by disomy 3, 6p gain, EIF1AX mutation, and Class 2 or B, characterized by disomy 3, gains in 6p/8q, SF3B1/SRSF2 mutation. Both subgroups exhibit a moderate chance of acquiring MUM at a subsequent developmental stage. On the other hand, Class 3 or C—defined by 8q gain, SF3B1/SRSF2/BAP1 mutations, and monosomy 3—and Class 4 or D—defined by multiple chromosomal copies, BAP1 mutations, 8q gain, and/or monosomy 3—are the subgroups with a bad prognosis [25]. The classifications derived from DNA methylation patterns, mutations, and/or chromosomal rearrangements have been shown to possess prognostic relevance. Consequently, they affect the tailored prognosis and therapeutic approaches in the management of UM.

### 3.1. GNAQ/11

The activation of the enzyme PKC (protein kinase C) and the signaling to the MAPK (mitogen-activated protein kinase) pathway depend on GNAQ/11 genes, which encode GTP-binding proteins.

Studies demonstrate that activating mutations in GNA11 and GNAQ, particularly at codons Q209 or R183 (inside the ras-like domain), occur in 85% to 94% of UM throughout all phases of the disease [26]. The aforementioned early-event changes are mutually exclusive and present in benign uveal nevi [27,28]. Their persistent activation of cell replication pathways, particularly MAPK/ERK, leads to abnormal cell division and tumor advancement, hence promoting tumor development and survival [29]. A recent finding uncovered the GNAQ hotspot mutation at codon G48, located in the phosphate-binding loop. [30]. The nucleotide-binding pocket is situated adjacent to the G48, R183, and Q209 mutations, as evidenced by recent structural analyses of active Gαq [31]. Consequently, mutations in G48, akin to those in Q209 and R183, may impede GTPase activity in a comparable manner. Recent discoveries have revealed further complexities in the functioning of GNAQ/11, encompassing the presence of several active domains of G proteins [32]. Furthermore, individuals with uveal melanoma (UM) exhibiting various GNAQ/11 mutations in their tumors, as determined by droplet digital PCR, demonstrated an increased probability of a poor outcome relative to those with homogeneous or absent mutations.

### 3.2. BAP1

Located on chromosome 3, the BAP1 gene has loss-of-function mutations that are connected to several types of cancers, including UM (subsets Classes 3/C or 4/D), which have a poor prognosis [33]. It is a constituent of the polycomb repressive deubiquitinase complex, which consists of ASXL1/2/3 and other combs such as 1/2/3. The main purpose of this complex is to remove monoubiquitin from H2AK119ub1 (a histone 2A protein that has been modified with a single ubiquitin molecule at lysine 119) [34]. In more than 40% of UM onsets, the absence of BAP1 provokes a state resembling stem cells, which can affect the differentiation of melanocytes and potentially facilitate the development of tumors in the other parts of the body [35]. The BAP1 protein has a complicated structure that contains many sections of inherently disordered proteins. These regions may help promote complex interactions and potentially affect disease processes. A recent epigenetic finding has revealed that there is a negative relationship between BAP1 expression and cg01493712 DNA methylation, which adds more complexity to our understanding of BAP1’s role [36].

### 3.3. PLCβ4

PLCβ4 is a crucial enzyme in cellular communication. It facilitates the degradation of phosphatidylinositol 4,5-bisphosphate (PIP2) into two significant molecules: inositol 1,4,5-trisphosphate (IP3) and diacylglycerol (DAG). The activation of PKC and the release of calcium from intracellular stores are contingent upon these mediators. [37]. Only a few cases of UM are diagnosed with a specific mutation in PLCβ4, located on codon D630. The PLCβ/ε, MAPK, and PKCδ/ε signaling pathways are constitutively activated by this mutation, which is categorized as an initiating mutation [37]. RasGRP3, a protein that facilitates the exchange of guanine nucleotides, is triggered into action by the PKC isoforms δ and ε. This activation triggers the activation of subsequent pathways, particularly the MEK/ERK axis, which has a crucial function in the development of UM tumors [38,39,40]. Nevertheless, the efficacy of PKC/MEK/ERK axis inhibitors in clinical settings is somewhat low. This suggests that PLCβ4 promotes the development of tumors through a different mechanism.

A recent study identified an active, nuclear-localized YAP1 (yes-associated protein 1) in the Tg(mitfa:PLCB4D630Y);tp53M214K/M214K;mitfa−/− zebrafish line; however, phosphorylated ERK, a marker of PLCβ signaling, was absent in these tumors [41].

### 3.4. CYSLTR2

Roughly 2–4% of instances of UM have been linked to the G-protein-coupled receptor CYSLTR2 [42]. The primary oncogenic event in cancers that have normal GNAQ and GNA11 genes is the recently identified particular mutation in codon L129. Mutations inGNA11, GNAQ, or PLCB4 have resulted in an increase in the frequency of the wild-type allele in UM tumors, while the amount of the mutant allele increased during tumor growth. This shows that these genetic changes have a complicated connection with one another [43].

### 3.5. SRSF2

SRSF2 is a component of the spliceosome, a complex that facilitates transcription elongation and preserves genomic integrity. Consequently, it contributes to the structural organization and regulation of alternative splicing in precursor mRNA [44]. A consideration of SRSF2 mutations in UM tumors revealed that a minority of patients displayed in-frame deletions at several protein residues [45]. Prognostic subgroups 2/B or 3/C exhibit these alterations. Because of these alterations, the mutant SRSF2 protein has a higher binding affinity for the CCNG nucleotide sequence than it does for the GGNG sequence, which alters the rates of exon inclusion [45]. In contrast to other cancer types, mutations in splicing factors such as SRSF2 and SF3B1 in UM result in a widespread reduction in the expression of genes associated with malignancy [46]. The dysregulation of alternative splicing is acknowledged to contribute to heightened tumor heterogeneity, cellular plasticity, and modified metabolism, which can influence therapeutic responses.

### 3.6. SF3B1

In 15–20% of UM cases, somatic missense mutations in SF3B1 are identified within prognostic classifications Classes 2/B or 3/C [47]. The primary location of the 625-arginine-residue (R625) is at the 625th position. Other infrequent occurrences encompass lysine 666 (K666) [48]. The subunit 1 of the splicing factor 3b protein complex has been encoded by the SF3B1 gene. During pre-mRNA splicing, this element is crucial for the synthesis of canonical spliced transcripts. Mutations in the SF3B1 gene result in spliceosome complexes utilizing various recognition sites, leading to abnormal spliced transcripts [49]. The incidence of metastatic illness in UM patients (*n* = 143 participants) with SF3B1 mutations was observed at both early and late stages of diagnosis. The disease was classified as occurring either prior to or subsequent to a follow-up period of 60 months, designated as early (under 60 months follow-up) and late stage (beyond 60 months) [50]. Previous studies have demonstrated that UM cases with mutations in BAP1 and SF3B1 are mutually exclusive. Nevertheless, the concurrent occurrence of SF3B1 mutation and BAP1 deficiency in UM cells induces senescence due to a diminished capacity to respond to DNA damage [51]. This indicates the potential for a synthetic lethal interaction contingent upon the genetic and epigenetic context. In UM malignancies, SF3B1 mutations induce alterations in the splicing mechanism. These alterations generate tumor neoepitopes that can alone be recognized by MHC class I and the patient’s CD8+ T-cells [52]. Also identified as possible anticancer possibilities are neoepitopes derived from the SF3B1-independent alternative splicing isoforms AMZ2P1 and MZT2B. The presence of these neoepitopes, when exposed to CD8+ T-cells, led to heightened IFN-γ production and caused cell death in UM cells [53]. Recent evidence indicates that the dysregulation of alternative splicing is a prevalent characteristic of malignancies, with significant therapeutic implications for their diagnosis, prognosis, and treatment. Short exons are more susceptible to dysregulation, irrespective of the cancer type. A panel of short exons associated with cancer demonstrated reliability as a predictor of survival across several cancer types [53].

### 3.7. MAPKAPK5

MAPKAPK5 (p38-regulated and activated kinase) is a type of serine/threonine protein kinase that is activated via the MAPK pathway. It is accountable for the commencement and control of a wide range of cellular activities, including apoptosis, gene expression, differentiation, and proliferation [54]. The mutation is found in around 2% of instances with UM. The TCGA analysis identified two primary alterations at residues Q473Nfs* and E106Kfs*23: the frameshift mutation of glutamine at position 473, which leads to the insertion of a premature stop codon, and the frameshift mutation of glutamic acid at position 106, which results in a sequence of 23 altered amino acids before encountering a premature stop codon. Nevertheless, there is a dearth of research on the impact of these mutations on UM [55].

### 3.8. EIF1AX

The X chromosome contains the EIF1AX gene, which codes for eukaryotic translation initiation factor 1A. This factor is important for the creation of 43S pre-initiation complexes, which are necessary for protein synthesis [56]. EIF1AX mutations are commonly found in UM, occurring in 14–20% of all cases, specifically in prognostic grouping 1/A. This indicates that EIF1AX plays a significant role as an oncogenic factor in UM. Mutants in exons 1 and 2 have been shown to boost total protein synthesis, which is consistent with the heightened demand for protein synthesis that is often observed in cancer cells [57].

**Table 1:** Most involved genes in progression of uveal melanoma: genotype–phenotype correlations and biochemical actions.

Gene	Name	Genetic Alterations	Phenotype	Action
GNAQ/11	G protein subunits alpha q/11	gene mutation	first genetic changes in uveal melanoma development [30]	activation of the enzyme protein kinase C and transmission of signals to the mitogen-activated protein kinase pathway
BAP1	BRCA1-associated protein 1	gene mutation; 8p gain; monosomy 3; 1p/8q gains	severe prognosis; higher metastatic risk [35]	loss-of-function mutations; constituent of the polycomb repressive deubiquitinase complex
PLCβ4	phospholipase C beta 4	gene mutation	first genetic changes in uveal melanoma development [39]	activation of the protein kinase C; activation and calcium release from intracellular reserves
CYSLTR2	cysteinyl-leukotriene receptor 2	gene mutation	first genetic changes in uveal melanoma development [44]	linking to the G-protein-coupled receptor; taking part in primary oncogenic events
SRSF2	serine-and arginine-rich splicing factor 2	gene mutation; 6p and 8q gains; monosomy 3; 1p/8q gains	intermediate prognosis [46]	arrangement of the structure and controlling of alternative splicing in precursor mRNA
SF3B1	splicing factor 3B subunit 1	gene mutation; 6p and 8q gains; monosomy 3; 1p/8q gains,	intermediate prognosis [50]	during pre-mRNA splicing, trigger of the synthesis of canonical spliced transcripts
MAPKAPK5	MAPK-activated protein kinase 5	unclassified mutation	highlight the genetic diversity of the uveal melanoma [56]	controlling gene expression, apoptosis, differentiation, and proliferation
EIF1AX	eukaryotic translation initiation factor 1A X-linked	gene mutation; 6p gain	less aggressive form of uveal melanoma [58]	involved in the protein synthesis; significant role as an oncogenic factor

## 4. Chromosomal Abnormalities

**Figure 1:**
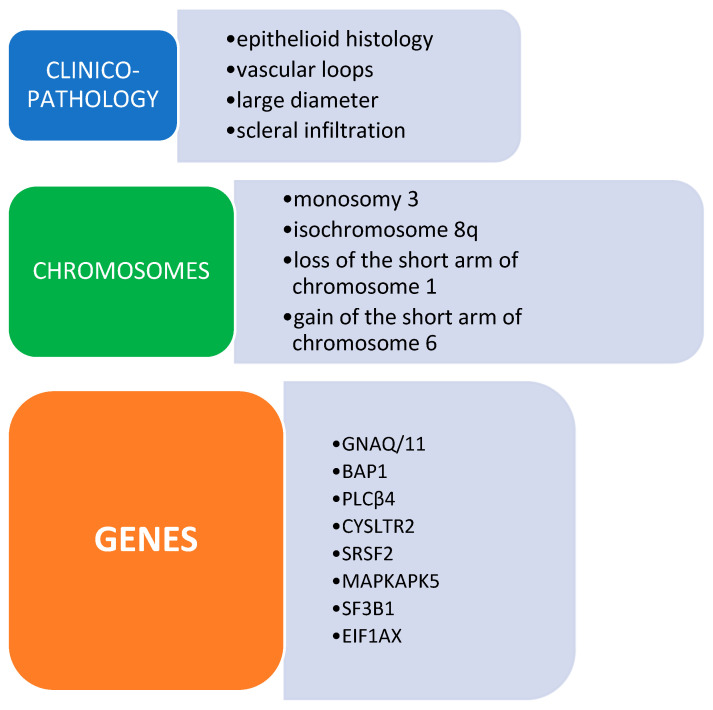
Clinico-hystological and genetic features of uveal melanoma.

The extent of genetic modifications caused by copy number changes (CNAs) varied significantly among patients, with a range of 0 to 53%. Chromosomal alterations are well recognized as important indications of prognosis and risk assessment in UM. Out of them, the absence of chromosomes 3, 8p, and 1p is a separate indicator of the distant spread of cancer [58]. Specifically, people with a high risk of metastases often show a condition called monosomy 3 and have higher tumor sizes. These features help us better understand the genetic causes that can lead to a more severe progression of the disease [59]. Recent breakthroughs in the research of low-frequency CNAs have shown a more detailed understanding of the genomic landscape (Figure 1).

Furthermore, the main focus of cytogenetic rearrangements in UM has been chromosomes 1, 3, 6, and 8. Monosomy 3 is frequently associated with isochromosome 8q and is observed as an early event in nearly 60% of the malignancies that have been examined. Isochromosome 8q is susceptible to aberrant segregation during mitosis, resulting in elevated levels of 8q gain. The literature has extensively analyzed the correlation between the risk of metastasis and these cytogenetic rearrangements, as follows in more detail [60].

### 4.1. Monosomy 3

The development of metastases and clinicopathologic features that suggest a poor prognosis, such as ciliary body involvement, high mitotic rate, large diameter, vascular loops, epithelioid histology, and extra-scleral extension, have been strongly associated with monosomy 3. Metastases are not common in tumors correlated to disomy 3 [61]. The 3-year prognosis of patients with UM who have complete monosomy 3, as assessed by fine-needle aspiration biopsy (FNAB), is worse than those with partial monosomy 3 or disomy 3 [62]. The cumulative likelihood of metastasis at 3 years is almost 3% for disomy 3, 5% for partial monosomy 3 (equivocal monosomy 3), and roughly 25% for complete monosomy 3. The clinical prognosis of patients with partial monosomy 3 or disomy 3 is not significantly different [63]. Moreover, when chromosome 3 has a normal copy number, tumors might display additional chromosome alterations, such as a 6p gain and a 1p loss [64].

### 4.2. Isochromosome 8q

Mutations in chromosome 8 are also prevalent in UM. The long arm of chromosome 8 (8q) is acquired in 38% to 65% of primary UM, a disorder that frequently derives from the isochromosome formation. A bleak prognosis is often associated with this condition [65,66]. Higher metastatic rates are associated with the coexistence of 8q gain and monosomy 3 [67]. According to reports, the 5-year mortality rate is nearly 70% in cases of concomitant monosomy 3 and 8q gain, while the mortality rates are roughly 40% and 30% in cases of monosomy 3 and 8q gain, respectively [68]. The starting chromosomal modification in the complex process of malignant transformation of UM has not yet been thoroughly investigated. According to some authors, monosomy 3 is the initial phase, whereas 8q gain occurs subsequently. Nevertheless, it has been emphasized by other authors that 8q gain occurs before chromosome 3 loss [69]. Finally, others reported that the telomeric portion of 8q is expanded in more than 90% of the examined UMs, indicating that it likely plays a crucial role in the development of UM tumors [70].

### 4.3. Deletion of the Short Arm of Chromosome 1

The deletion of the short arm of chromosome 1 (1p) is commonly linked to monosomy 3 in 19–34% of uveal melanoma (UM) cases and in 33% of metastatic malignancies [71]. The simultaneous loss of 1p and monosomy 3 has been identified as an independent predictive factor for disease-free survival [72].

### 4.4. Amplification of the Short Arm of Chromosome 6

The incorporation of the short arm of chromosome 6 (6p) was the inaugural chromosomal aberration documented in uveal melanoma (UM). The occurrence of this mutation in UM ranges from 18% to 54% and is associated with a positive outcome [71,72].

## 5. DNA Methylation

DNA methylation, a prevalent form of epigenetic modification, modulates gene expression via two distinct mechanisms: it can obstruct the activity of transcriptional proteins, hindering their binding to the gene, or it can engage with methyl-CpG-binding domain proteins, leading to the aggregation of inactive chromatin. Both strategies exemplify epigenetic changes.

The methylation of BAP1 at a particular genomic locus is connected to BAP1 protein levels, BAP1 mutations, and BAP1 genomic copy loss, according to a study by Bakhoum [73,74]. The researchers determined that this provides valuable predictive information, even in tumors where whole-exome sequencing was unable to detect any BAP1 alterations. Additionally, they found that the spread of UM to other parts of the body is linked to the deletion of BAP1 in the original tumor. Aberrant methylation of BAP1 and SF3B1 was discovered to be the cause of UM metastases in the bone, skin, and liver. The research showed a high degree of patient diversity and that several tumor-related genes are altered as a result of epigenetic changes in metastasized UM [75]. Through the utilization of integrated differential DNA methylation and gene expression analysis, researchers identified genes that are associated with early metastasis and a negative prognosis. TMEM200C, RGS10, ADAM12, and PAM are genes that are undermethylated and have been identified as potential oncogenes associated with early metastasis. On the other hand, RNF13, ZNF217, and HYAL1 are genes that are over-methylated and have been identified as potential tumor suppressors [76]. The decreased expression of tumor suppressor genes, specifically p16INK4a, RASSF1A, and p16INK4b, may have a major impact on the development of tumors. Hypomethylation of the tumor suppressor gene occurs less frequently. Nevertheless, it has been found that the DSS1 gene and preferentially expressed antigen in melanoma (PRAME), which serves as a dependable marker for metastasis, are excessively expressed in UM due to hypomethylation [77].

A recent study conducted in Slovakia analyzed tissue samples from 68 individuals with UM. The findings showed that high-risk tumors exhibited about 7810 CpGs that had reduced methylation (hypomethylation) and 16,588 CpGs that had increased methylation (hypermethylation). Three genes, namely, AHNAK2, HTR2B, and CALHM2, showed upregulation or hypomethylation, whereas six genes, namely, EDNRB, SLC25A38, RNF43, TLR1, IL12RB2, and MEGF10, showed downregulation or hypermethylation. These genes were detected for validation, and the number of CpGs showed an inverse correlation with gene expression [78].

## 6. Genes and Biochemical Pathways Related to Metastatic Disease

The conversion from epithelial to mesenchymal tissue occurs during the advanced stages of UM. The epithelial–mesenchymal transition (EMT) is a group of events that cause cells to lose their adherent junctions and assume a highly mobile fibroblastoid phenotype, which is spindle-shaped. Many transcription factors may regulate the process of EMT. These factors play a role in the progression of cancer and the metastasis of cancer cells in different types of tumors. The function of transcription factors in UM, however, is not well understood. Initial investigations have indicated that the suppression of ZEB1, Twist1, and Snail1 reduces the invasive traits of UM cells. Conversely, increased mRNA levels of Twist1 as well as ZEB1 are linked to a more aggressive phenotype of malignant UM [79]. Previous studies have emphasized that the process of EMT in UM cells might be triggered by prolonged exposure to the pro-inflammatory cytokine IL-6. The IL-6/STAT3 signaling pathway triggers the activation of JunB, leading to the occurrence of EMT alterations. Thus, the IL-6/STAT3/JunB pathway significantly promotes the migration and invasion of UM cells [80].

Novel genes, including those associated with pathogenesis, have been found in the metastatic course of uveal melanoma as it genetically evolves from primary tumor to metastatic illness. Several investigators have identified multiple genes involved in the process of EMT and have found a connection between these genes and the existence of monosomy 3.

The CCL18 gene exhibited the most significant upregulation. Tumor-infiltrating lymphocytes (TILs) are primarily produced by CCL18, which also has chemotactic activity toward naive T-cells, CD4+, and CD8+ T-cells. Unlike other types of cancer, the existence of tumor-infiltrating lymphocytes in UM has been linked to a negative outlook [81]. However, the precise mechanism by which they contribute to the advancement of the illness has not yet been determined.

UM cell movement in vitro and invasiveness in vivo are enhanced by high expression levels of PTP4A3/PRL-3, which is known to be strongly predictive of metastasis [82]. PTP4A3 functions both directly and indirectly via the membrane accumulation of MMP14, a membrane-anchored metalloprotease that is essential for the remodeling, invasion, and turnover of the extracellular matrix (ECM) required for migration or invasiveness. This is a crucial meta-static event that is involved in the development of cancer [83]. Furthermore, PTP4A3 showed a correlation with the expression of several proteases such as ADAM10, which is well-known to be elevated in melanoma metastases, being associated with multiple adhesion molecules that play a crucial role in the development of malignant melanoma.

By employing 2D phosphoprotein analysis, scientists have discovered that CRMP2 is a new target for PTP4A3. The presence of CRMP2 is a favorable prognostic factor, suggesting a reduced risk of metastasis development, as it hinders the movement and infiltration of UM cells. PTP4A3 inhibits the expression of CRMP2 [84,85].

S100A4 is another gene that has a role in promoting movement and invasion. These genes have been associated with the formation of tumor metastasis [86].

Another gene that exhibits upregulated expression is the PRRX1 transcription factor. This transcription factor facilitates the molecular mechanism of EMT and augments the invasive capabilities of cells in the surrounding tissues.

Tumor invasion and metastasis can result from the multidirectional differentiation capability and self-renewal of cancer stem cells [61]. As a consequence of the EMT process, recent data suggest that the presence of cells with stem cell-like features plays a role in drug resistance [87]. There has been limited research examining the involvement of cancer stem cells in UM. The identification of globally accepted indicators for melanoma stem cells is still absent, and the understanding of their routes is still incomplete. While class 1 cancers exhibit resemblance to fully developed neural crest cells and differentiated melanocytes, class 2 tumors display transcriptional similarity to primordial neural and ectodermal stem cells. Class 2 tumors do not resemble undifferentiated embryonic stem cells, indicating that the class 2 hallmark represents the development of a specific basic transcriptional program for a particular lineage, rather than a general process of reverting to an undifferentiated state [88].

## 7. Proteins in Ocular Fluid as Biomarkers

Fluids located in front of and behind the lens, respectively, are called aqueous humor (liquid fluid) and vitreous humor (gel-like fluid). Aqueous humor comprises organic and inorganic ions, glutathione, carbohydrates, amino acids, carbon dioxide, oxygen, and water [89]. The assessment of numerous cytokines, chemokines, growth factors, and other proteins is crucial for diagnosing different ocular conditions, and all of these proteins are regarded as biomarkers for the diagnosis, prognosis, and therapy of UM.

Between Chinese patients with UM and control subjects with cataracts, significant differences were observed in the expression of numerous angiogenic, chemotactic, and inflammatory cytokines, such as IP-10, IL-6 and IL-8, NGF-β, PLGF-1, b-FGF, EGF, VEGF-A, and RANTES [90]. Comparable studies were conducted on 35 Italian individuals with UM and 35 cataract controls. When UM patients’ aqueous humor was compared to those of subjects with cataracts, the results revealed greater amounts of IL-6, IL-8, EGF, bFGF, macrophage inhibitory factor (MIF), and MCP-1 [91].

Researchers also looked into the protein makeup of the vitreous humor of UM patients. The comparison between Dutch patients with UM and controls indicated elevated vitreous fluid concentrations of IL-6, IL-8, IP-10, MCP-1, macrophage inflammatory protein 1α (MIP-1α), MIP-1β, TNF-α, and RANTES. Furthermore, it was demonstrated that the concentrations of IL-6, IL-8, MCP-1, IP-10, MIP-1β, MIP-1α, TNF-α, GCSF, RANTES, IFN-γ, and VEGF are positively correlated with tumor size [92]. Significantly elevated concentrations of angiogenin, IL-8, and MCP-1 were observed in the aqueous humor of Japanese subjects with UM in comparison to those with certain benign ocular tumors. The data suggest that IL-8, MCP-1, and angiogenin may function as possible biomarkers for distinguishing malignant UM from benign intraocular malignancies [93].

Compared to subjects with a cataract, Austrian patients with UM exhibited higher concentrations of Flt-3 ligand, IL-6, IL-8, (IP)-10, MCP-1, MIP-1α, platelet-derived growth factor AA (PDGF-AA), and VEGF in their vitreous and aqueous humor in biomarker measurements. Patients with UM had considerably greater levels of eotaxin in their aqueous humor and IL-7 in their vitreous humor. Tumor dimensions exhibited strong correlations with IP-10 and MIP-1 in both aqueous and vitreous humor, but Fms-related tyrosine kinase 3 ligand (FLT3LG), IL-6, IL-8, and MCP-1 showed positive relationships solely in vitreous humor. A positive correlation was observed between FLT3LG and MCP-1 (in aqueous and vitreous humor) and IL-8, IP-10, MIP-1α, and PDGF-AA (in vitreous humor) in the context of tumor infiltration in Bruch’s membrane, an extracellular matrix that lies between the retinal pigment epithelium and the choroid [94].

Dutch patients with UM underwent investigations on soluble human leukocyte antigens (sHLA). Metastases were more prevalent in patients with sHLA-positive aqueous humor, and their survival was substantially reduced [95].

In summary, many case–control studies examining uveal melanoma and benign ocular illnesses have identified angiogenic, inflammatory, and chemotactic indicators as critical elements linking inflammation to carcinogenesis.

Besides the diagnostic role of cytokines and growth factors, biomedical research yields biomarkers that are valuable for assessing therapy efficacy and prognostic significance.

## 8. Conclusions

Recent studies have found polymorphisms in the BARD1 and BRIP1 genes in patients with UM and atypical choroidal nevus (i.e., pigmented/non-pigmented choroidal formation with signs of growth within 2 years of observation) [96]. At present, researchers are examining these findings to assess the risk levels, methods of prevention, and methods of diagnosing intraocular neoplasms and UM. Additionally, a specific case study has highlighted the complex genetic characteristics of UM by revealing the presence of many mutations, including a specific mutation in the PBRM1 gene, that is responsible for encoding the BAF180 protein involved in the chromatin remodeling process [97]. Furthermore, it has been discovered that the LRP1B and CHEK2 genes exhibit mutations in UM samples, potentially indicating a connection with high-risk characteristics [98]. Advancements in the molecular knowledge of UM are aiding in the development of more accurate diagnostic, prognostic, and treatment methods. The identification of particular mutations, chromosomal abnormalities, and anatomical variations within different subtypes of UM is contributing to the development of a more individualized approach to therapy. Additional intricacies have been discovered in the composition and control of BAP1, and initial stages in mutations of GNAQ/11 have been broadened to encompass the understanding of active conditions. As efforts to better understand the etiology of UM have turned to genetic research, relevant chromosomal abnormalities and genetic mutations have been found that may be effectively used as biomarkers to forecast prognosis. Genetic biomarkers are now the most dependable approach for outcome detection, regardless of the methodology used; however, no single approach is absolutely perfect. This review summarizes our current knowledge about UM genetics and analyzes how molecular and microbiological changes function as catalysts, and most crucially, how their actions might be inhibited. It is quite possible that further research will be able to clarify the connection between certain genetic alterations and their function in UM in this regard. It is a whole new ball game to understand how large chromosomal alterations, like those of 1p, M3, 6p+, and 8q+, function to drive or differentiate UM behavior. Additionally, a largely uncharted territory that may influence these alterations is beginning to take shape. According to recent research, the well-characterized genetic markers of UM may merely serve as markers for epigenetic modulators that have the power to further subdivide the broad-brush genetic classes into more focused groups, with implications that are still being worked out. The genomic framework of UM appears to be significantly more complex than previously believed, despite not being as clearly unstable as many malignancies.

## Data Availability

No new data were created.
